# Accessing the Levator Labii Superioris Alaeque Nasi (LLSAN) Muscle for the Treatment of Infraorbital Dimples—A Case Report

**DOI:** 10.1111/jocd.70534

**Published:** 2025-11-24

**Authors:** Sergio Escobar, Maria Victoria Silva Oliveira, Mariana Calomeni, Carlos Bravo, Paula Arroyo, Pamela Saavedra, Sebastian Cotofana, Michael Alfertshofer

**Affiliations:** ^1^ Private Practice Buenos Aires Argentina; ^2^ Clinica Bravo Rio de Janeiro Brazil; ^3^ Private Practice San José Costa Rica; ^4^ Private Practice Santiago Chile; ^5^ Department of Dermatology Erasmus Medical Centre Rotterdam the Netherlands; ^6^ Centre for Cutaneous Research, Blizard Institute Queen Mary University of London London UK; ^7^ Department of Plastic and Reconstructive Surgery Guangdong Second Provincial General Hospital Guangzhou Guangdong Province China; ^8^ Department of Oral and Maxillofacial Surgery Charité University Hospital Berlin Berlin Germany

**Keywords:** facial aesthetics, facial anatomy, infraorbital dimple, LLSAN muscle, muscular balance

## Abstract

**Background:**

Medial infraorbital dimples are dynamic soft tissue depressions that can emerge or intensify following neuromodulator treatment in the periorbital region. Rather than being congenital or structural in origin, these dimples often result from imbalanced facial muscles most notably the levator labii superioris alaeque nasi (LLSAN).

**Objective:**

To elucidate the pathophysiology of a medial infraorbital dimple arising after botulinum toxin injection into the lateral orbicularis oculi muscle (OOM) and to demonstrate the clinical efficacy of a targeted neuromodulator injection into the LLSAN muscle.

**Case Summary:**

We present a case involving a 31‐year‐old female who developed a prominent medial infraorbital dimple four weeks after receiving a cosmetic botulinum toxin injection for her crow's feet. This dimple was most prominent during smiling. A single, superficial injection of 2 units of botulinum toxin type A into the LLSAN led to substantial aesthetic improvement, starting 2 days after the treatment with preserved natural expression and no functional deficit.

**Conclusion:**

Recognition of compensatory muscular activity and intramuscular injection of neuromodulators for the LLSAN offers a safe and effective approach for treating medial infraorbital dimples while preserving facial harmony.

## 1 Introduction

Infraorbital dimples that appear or become more pronounced with facial expression, particularly smiling, are most often dynamic rather than structural in nature. These soft tissue concavities typically result from imbalances in mimetic muscle activity, which may arise spontaneously or be induced by neuromodulator treatments [1].

In particular, treatments targeting the lateral periorbital region—for example, during the treatment of lateral canthal lines that are also termed crow's feet—can unintentionally disrupt the delicate equilibrium of facial muscle groups [2, 3]. In some individuals, this may lead to compensatory activity of untreated muscles, such as the levator labii superioris alaeque nasi (LLSAN). The resulting shift in facial dynamics can produce a visible dimple inferior to the tear trough during smiling, often misattributed to congenital or structural causes [4].

Although such changes are subtle, they can significantly affect perceived facial symmetry and patient satisfaction. Yet they remain underrecognized in clinical practice.

This report presents a case of a treatment‐induced medial infraorbital dimple attributed to presumed LLSAN activity, which was effectively treated using neuromodulator injections. The case highlights the importance of functional muscle assessment in aesthetic medicine [5].

## 2 Case Presentation

A 31‐year‐old female presented to our clinic with the primary complaint of a newly visible dimple in her medial infraorbital region, which had appeared 4 weeks after receiving botulinum toxin injections for the treatment of her lateral canthal lines in the lateral orbicularis oculi muscle (OOM; 15 I.U.; onabotulinumtoxin A, on‐label injection technique). The dimple was especially prominent during smiling and emotional expression. The patient denied any prior history of congenital dimples, facial trauma, or surgical procedures in the midface region. Written informed consent was obtained from the patient for the diagnostic procedures, treatment, and publication of this case report, including the use of clinical images.

**FIGURE 1:**
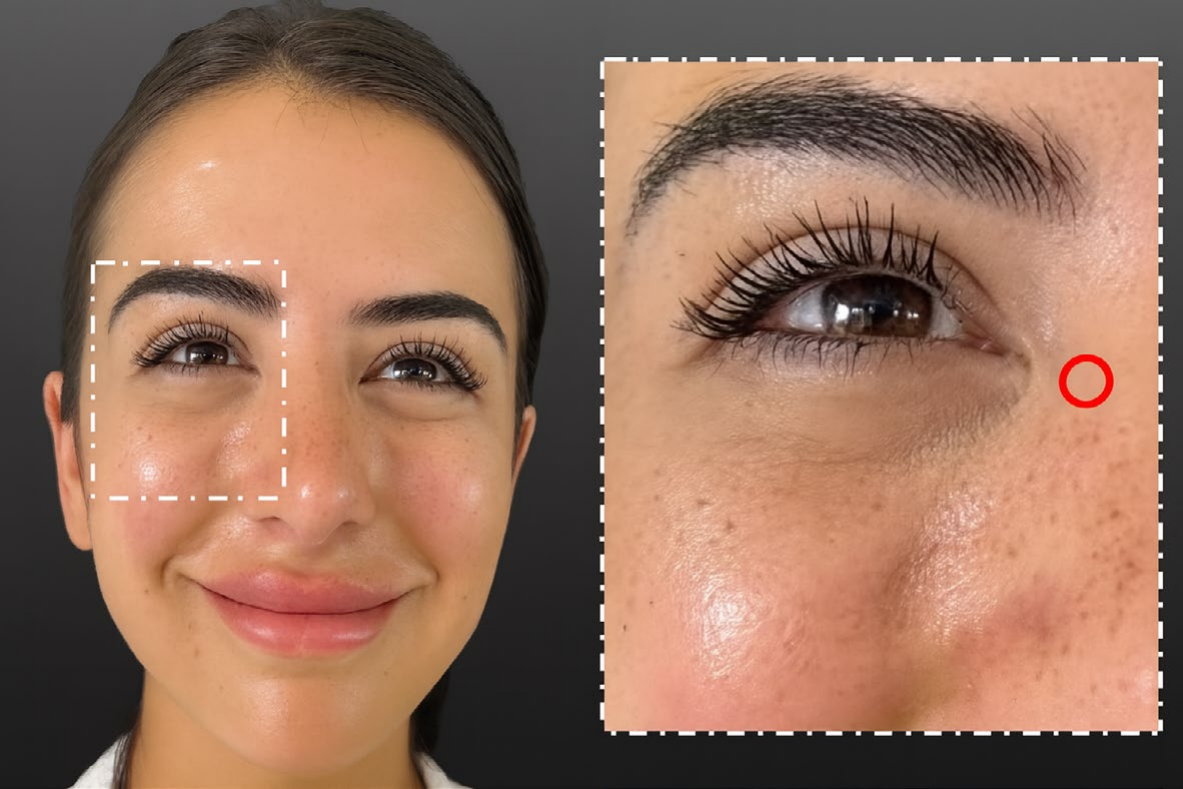
Pretreatment photograph of the 31‐year‐old female patient showing a well‐demarcated medial infraorbital dimple upon smiling, four weeks after neuromodulator injection into the lateral orbicularis oculi muscle (OOM) for crow's feet. The red circle marks the injection location.

Clinical examination revealed a prominent concavity located medial to the midpupillary line, superior to the nasolabial fold, and approximately 1 cm inferior to the tear trough. The dimple was not visible at rest but became prominent with smiling, underlining the dynamic rather than structural etiology. No signs of asymmetry, scarring, or dermal tethering were present upon manual examination (Figure 1).

## 3 Ultrasound Imaging

**FIGURE 2:**
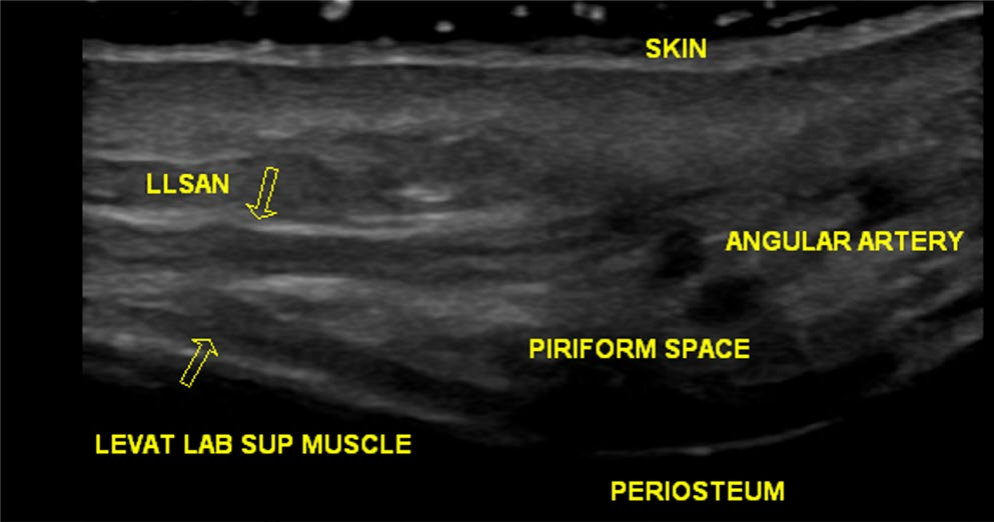
High‐frequency ultrasound image of the same patient's medial infraorbital region, showing the underlying muscular anatomy, including the LLSAN.

To support the clinical diagnosis and ensure precise injection placement, ultrasound imaging (Logiq E ultrasound, General Electric, Milwaukee, WI, USA; 18 MHz linear probe; hockey stick) was used. The LLSAN was observed to be a thin, hypoechoic structure emerging from the periosteum of the frontal process of the maxilla inferiorly toward its insertion at the upper lip and nasal ala, consistent with known anatomy [6] (Figure 2). Dynamic ultrasound investigation during smiling confirmed intact contractile activity of the LLSAN but reduced activity of the laterally located OOM. This imaging presentation confirmed the previous treatment algorithm in which the lateral OOM was relaxed during the treatment of lateral canthal lines, but the LLSAN was not injected. No asymmetrical activity was detected, ruling out residual or patchy neuromodulator effect.

**FIGURE 3:**
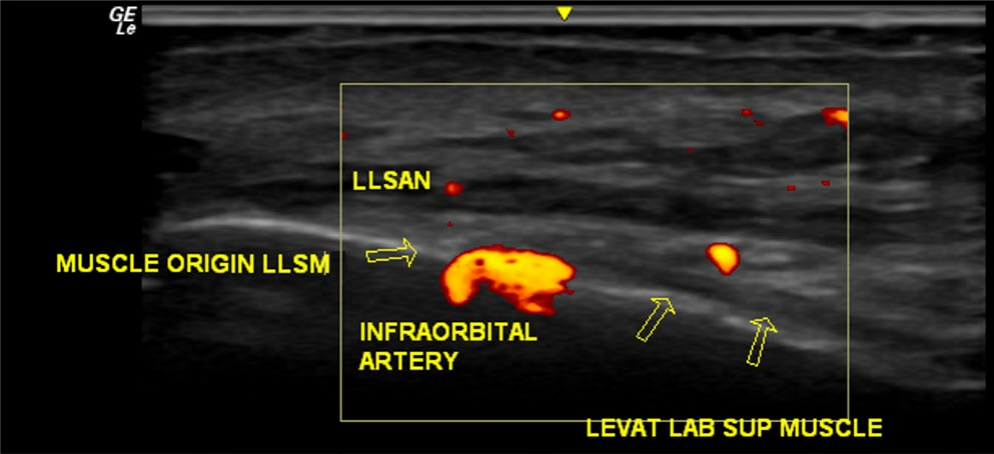
High‐frequency ultrasound image of the same patient's medial infraorbital region, showcasing the anatomy of the infraorbital artery in relation to the surrounding facial muscles.

Ultrasound also enabled the identification of critical vascular structures, including the angular artery and infraorbital vessels, allowing for safe planning of the injection route. This imaging step was essential to ensure precise deposition of the neurotoxin while avoiding spread to nearby functional muscles (Figure 3).

## 4 Case Management

A targeted injection of 2 units of botulinum toxin type A (onabotulinumtoxin A, 1.0 cc off‐label reconstitution volume) was administered superficially into the LLSAN belly via a 31G 4‐mm needle. The injection was administered to the dimple medially targeting the superficially located LLSAN. Key safety measures during the procedure included the following: (i) avoidance of the angular artery, which was located deep; (ii) a single‐point injection with low volume to minimize unwanted spread; (iii) postinjection compression to reduce the risk of bruising or hematoma formation. The therapeutic goal was to reduce excessive LLSAN contraction without compromising upper lip elevation or nasal function, thereby restoring facial harmony.

**FIGURE 4:**
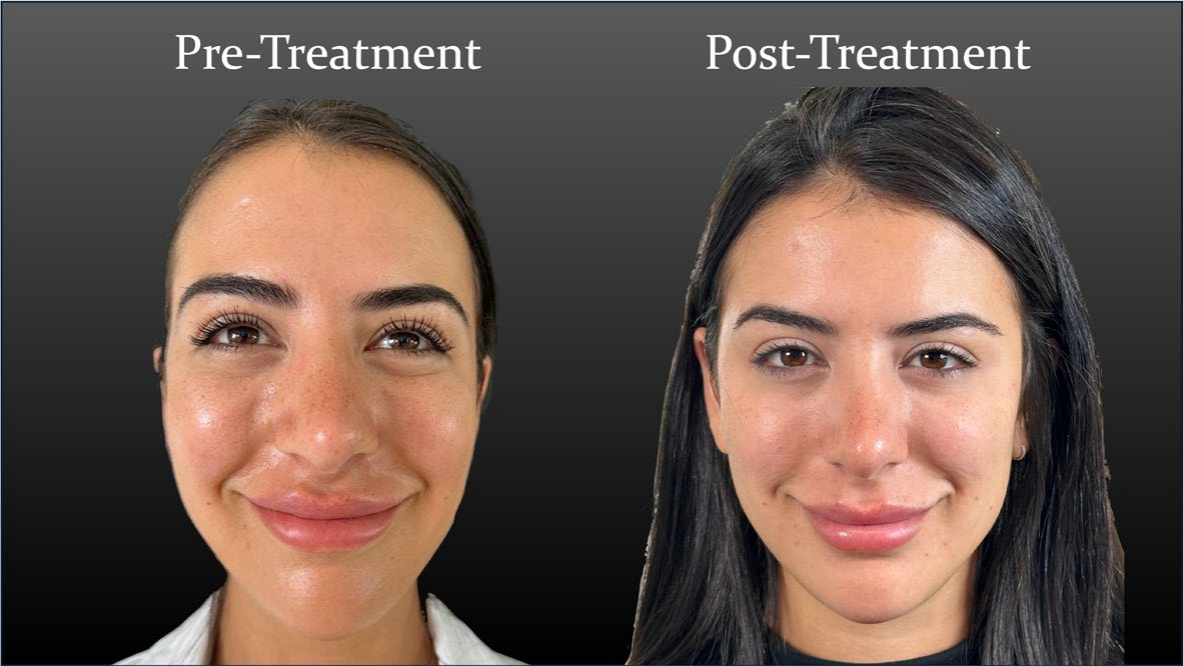
Side‐by‐side comparison of pre‐ and posttreatment photographs during smiling. The posttreatment image shows complete resolution of the dynamic infraorbital dimple following injection into the LLSAN.

After a two‐day follow‐up, the dimple disappeared, and the patient reported a noticeable aesthetic improvement in her infraorbital dimple during smiling. Our clinical evaluation confirmed complete resolution of dynamic dimpling in the medial infraorbital region while preserving remaining facial expressions without signs of an asymmetrical smile. Further, no other adverse effects were reported. These results were sustained at a 12‐week follow‐up, with no evidence of recurrence or compensatory overactivity in neighboring muscles (Figure 4).

## 5 Discussion

LLSAN is a vertically oriented muscle that originates from the frontal process of the maxilla in the close proximity to the medial canthal ligament. In that specific location it shares its bony and ligamentous origin with the OOM. It travels as a thin muscular band inferiorly and serves as a connection between nasalis and medial OOM fibers [6]. Although the OOM becomes incorporated into the midfacial superficial musculo‐aponeurotic system (SMAS), the LLSAN travels deep and connects with the levator labii superioris (LLS) before inserting into the nasal ala and then into the upper lip. Upon its fusion with the LLS, the LLSAN and the LLS connect with the OOM of the perioral region [7].

**FIGURE 5:**
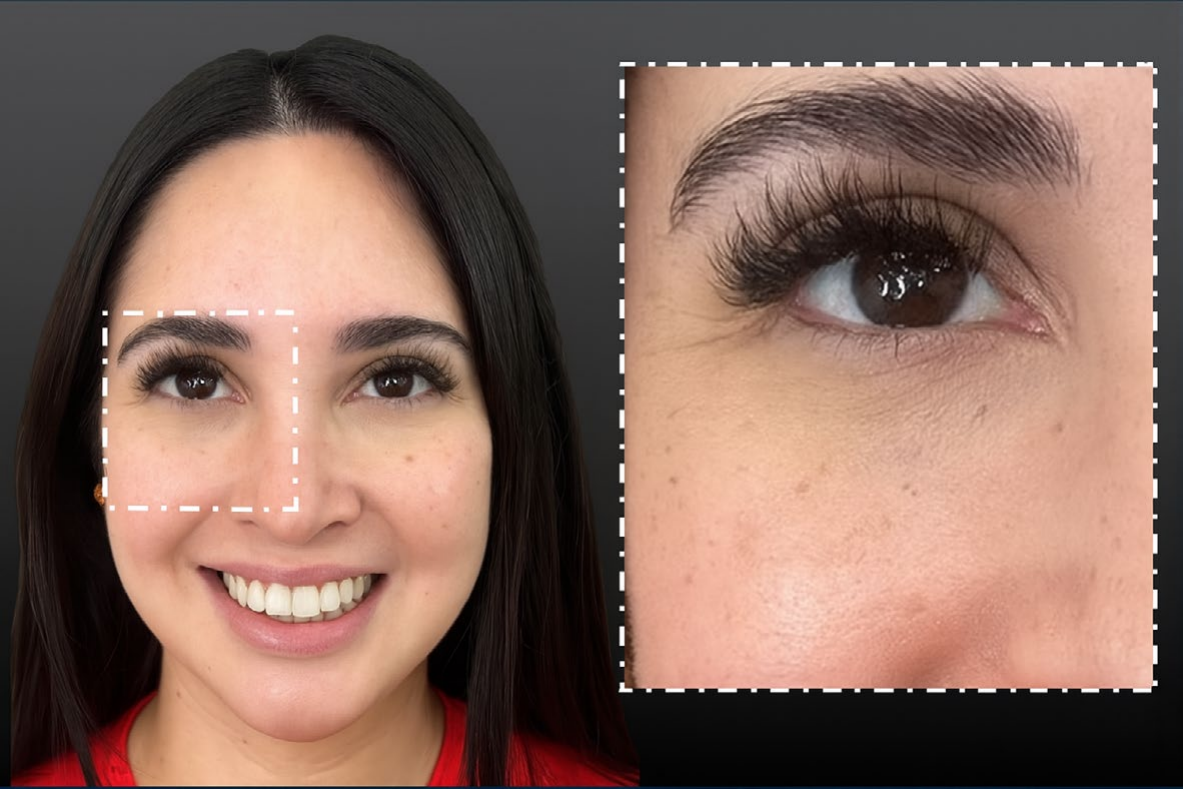
Posttreatment photograph of the same patient during a non‐Duchenne smile, demonstrating natural expression and the absence of infraorbital dimpling after targeted injection of 2 units of onabotulinumtoxin A into the LLSAN belly.

**FIGURE 6:**
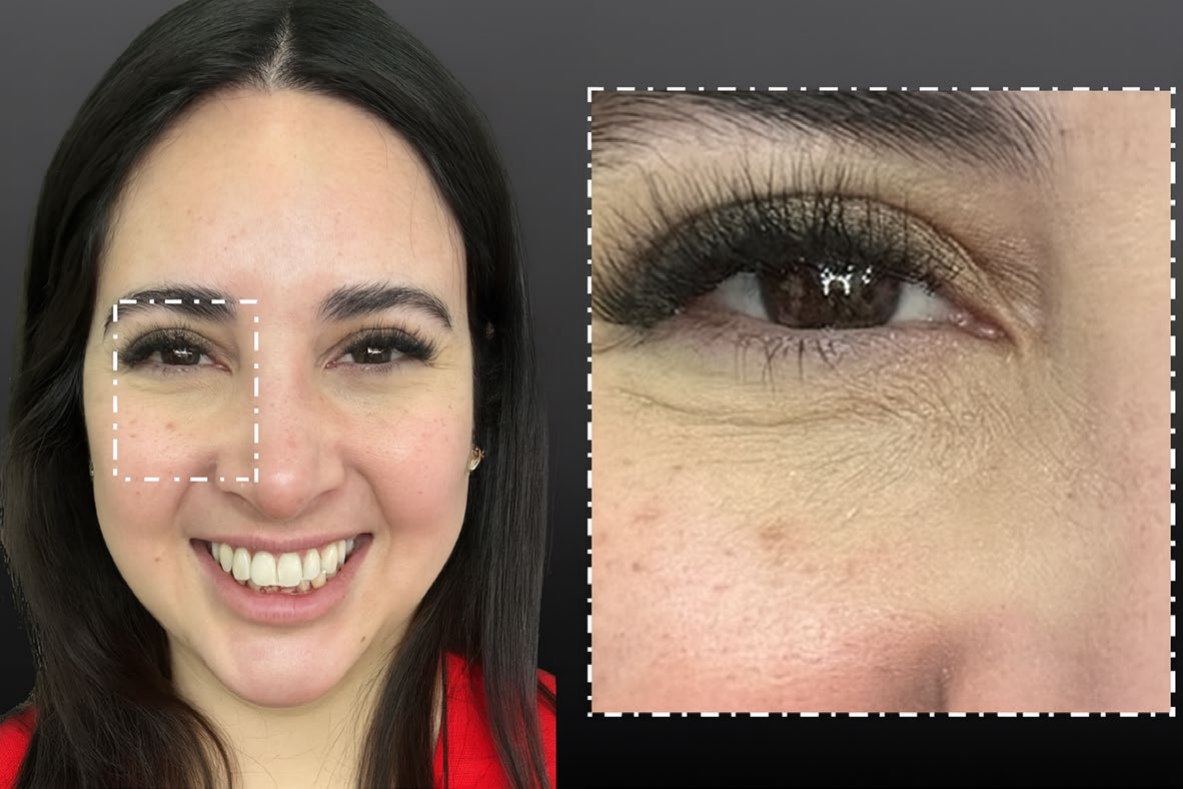
Posttreatment photograph of the same patient during a Duchenne smile, confirming preserved periorbital dynamics and full resolution of the infraorbital dimple after targeted injection of 2 units of onabotulinumtoxin A into the LLSAN belly.

In its anatomic function, the LLSAN serves as an upper lip elevator, thereby contributing to deepening the nasolabial fold, elevating the medial lip, and flaring the nostril [8]. In its upper third, the LLSAN is connected to the OOM, where both muscles form a continuous muscular plate. Though these muscles serve different functions—periorbital closure versus lip elevation—they often co‐contract during expressive movements such as smiling, crying, or squinting, underlining their finely adjusted activity in various facial expressions [9, 10]. This coordinated activation forms part of a broader network of mimetic muscle synergy across the midface (Figures 5 and 6).

In the case presented, botulinum toxin was used to weaken the lateral fibers of the OOM to treat lateral canthal lines. This intervention altered the balance of muscular tension in the midface. With reduced contractility in the OOM, compensatory recruitment of synergistic or adjacent muscles becomes more pronounced during animation. The LLSAN, particularly its superior portion near the orbital rim, responded with activation, whereas directly lateral to it, the OOM was relaxed without muscular tone. The resulting dynamic tension along its vertical vector created a visible, animation‐dependent tethering effect in the medial infraorbital region—clinically manifesting as a dimple. This phenomenon is facilitated by the thin soft tissue coverage in the medial midface, which especially in Asian patient populations results in the presence of the midfacial dimple.

From a therapeutic standpoint, the challenge lies in targeting the LLSAN exclusively without affecting adjacent muscles like the LLS, ultimately causing unnatural facial expressions. The LLSAN is tightly connected—both anatomically and functionally—with other perioral muscles such as the LLS and the levator anguli oris muscle (LAO) [11]. Overtreatment can result in unnatural flattening of the nasolabial fold, smile asymmetry, or unintended effects on speech and phonation. In the present case, a targeted injection into the LLSAN was administered resulting in complete resolution of the dynamic dimple without compromising lip function or smile aesthetics. This was achieved by a superficial injection in which the needle tip was advanced medially and not deeply; this is especially important to avoid the LLS and the LAO from getting affected. The immediate disappearance of the dimple beginning 2 days after the treatment shows that the correct muscle was targeted. The absence of any side effects additionally confirms that only the LLSAN was targeted and not the LLS or the LAO.

Ultimately, this case illustrates how minor yet visually impactful complications can arise from well‐executed aesthetic treatments due to the interconnectedness of facial muscle groups. It reinforces the importance of understanding not only static anatomy but also the dynamic relationships responsible for various facial expressions. Thorough anatomical knowledge remains the foundation of safe aesthetic interventions; in selected cases, the integration of functional imaging—particularly ultrasound—can serve as a valuable adjunct to confirm anatomy, assess muscle dynamics, and visualize vascular structures, especially in patients with indistinct surface landmarks or thin soft tissue coverage.

## 6 Conclusion

Medial infraorbital dimples may emerge or become more pronounced due to compensatory LLSAN activity following periorbital neuromodulator treatments. Superficial botulinum toxin injections into the LLSAN muscle represent a safe, effective, and targeted treatment option. A detailed understanding of facial muscle anatomy and biomechanics enables precise aesthetic interventions that preserve natural expression while addressing patient concerns.

## Author Contributions

S.E. and M.V.S.O. were the primary physicians involved in patient diagnosis, clinical care, and follow‐up. M.C. and C.B. supported clinical evaluation and contributed to the critical interpretation of clinical findings. M.V.S.O, P.A., and P.S. assisted in anatomical localization, patient communication, and documentation of photographic and imaging data. S.C., S.E., P.A., and M.A. provided anatomical expertise and contributed to the ultrasound‐based analysis and interpretation. S.E., C.B., M.C., and M.A. drafted the manuscript and coordinated literature review and figure preparation. S.C. and M.A. jointly revised the manuscript. All authors contributed to the conceptualization of the case report, critically revised the manuscript, and approved the final version.

## Conflicts of Interest

The authors declare no conflicts of interest.

## Data Availability

The data that support the findings of this study are available from the corresponding author upon reasonable request.

## References

[1] J. Pessa , V. Zadoo , E. K. Adrian, Jr. , C. H. Yuan , J. Aydelotte , and J. R. Garza , “Variability of the Midfacial Muscles: Analysis of 50 Hemifacial Cadaver Dissections,” Plastic and Reconstructive Surgery 102, no. 6 (1998): 1888–1893. 9810983 10.1097/00006534-199811000-00013

[2] M. A. C. Kane , S. E. Cox , D. Jones , X. Lei , and C. J. Gallagher , “Heterogeneity of Crow's Feet Line Patterns in Clinical Trial Subjects,” Dermatologic Surgery 41, no. 4 (2015): 447–456, 10.1097/DSS.0000000000000336. 25789814

[3] J. Z. Piao , W. Oh , Y. J. Choi , et al., “Ultrasonographic Analyses of Crow's Feet and Novel Guideline for Botulinum Toxin Injection,” Journal of Cosmetic Dermatology 21, no. 9 (2022): 3787–3793, 10.1111/jocd.15167. 35716350

[4] H. J. Moon , W. Lee , and J. Y. Choi , “Dynamic Evaluation of Facial Muscles: 3D Skin Displacement Vector Analysis Using a Facial Painting Model,” Laryngoscope Investigative Otolaryngology 6, no. 4 (2021): 650–656. 34401486 10.1002/lio2.590 PMC8356875

[5] O. Guntinas‐Lichius , V. Trentzsch , N. Mueller , et al., “High‐Resolution Surface Electromyographic Activities of Facial Muscles During the Six Basic Emotional Expressions in Healthy Adults: A Prospective Observational Study,” Scientific Reports 6, no. 13 (2023): 19214. 10.1038/s41598-023-45779-9 PMC10628297 37932337

[6] M. Hur , K. Hu , J. Park , K. Youn , and H. Kim , “New Anatomical Insight of the Levator Labii Superioris Alaeque Nasi and the Transverse Part of the Nasalis,” Surgical and Radiologic Anatomy 32, no. 8 (2010): 753–756. 20512646 10.1007/s00276-010-0679-4

[7] M. S. Hur , S. Lee , H. S. Jung , and R. A. Schneider , “Anatomical Connections Among the Depressor Supercilii, Levator Labii Superioris Alaeque Nasi, and Inferior Fibers of Orbicularis Oculi: Implications for Variation in Human Facial Expressions,” PLoS One 17, no. 3 (2022): e0264148. 35231048 10.1371/journal.pone.0264148 PMC8887774

[8] C. C. Snider , A. N. Amalfi , L. E. Hutchinson , and N. Z. Sommer , “New Insights Into the Anatomy of the Midface Musculature and Its Implications on the Nasolabial Fold,” Aesthetic Plastic Surgery 41, no. 5 (2017): 1083–1090. 28508263 10.1007/s00266-017-0889-9

[9] N. Kitagawa , J. Iwanaga , R. S. Tubbs , H. Kim , Y. S. Moon , and M. S. Hur , “Variant Muscle Fibers Connecting the Orbicularis Oculi to the Orbicularis Oris: Case Report,” Anatomy & Cell Biology 55, no. 4 (2022): 497–500. 36044997 10.5115/acb.22.108 PMC9747335

[10] K. L. Lee and H. J. Kim , “Topographic Analysis of the Periorbital Region Including Orbicularis Oculi Muscle Based on Ultrasonography Interpretation,” Journal of Cosmetic Dermatology 24, no. 1 (2025): e70004. 39835471 10.1111/jocd.70004 PMC11748098

[11] M. S. Hur , J. O , H. M. Yang , et al., “Heights and Spatial Relationships of the Facial Muscles Acting on the Nasolabial Fold by Dissection and Three‐Dimensional Microcomputed Tomography,” PLoS One 15, no. 8 (2020): e0237043. 32750081 10.1371/journal.pone.0237043 PMC7402499

